# Supplementary material for: New Species of Large-Spored Alternaria in Section Porri Associated with Compositae Plants in China
Source: J Fungi (Basel). 2022 Jun 6;8(6):607. doi: 10.3390/jof8060607 (PMC9225545; doi:10.3390/jof8060607)
Supplement: Supplementary file 1 [file jof-08-00607-s001.zip › jof-1752105-Figure S1.pdf]

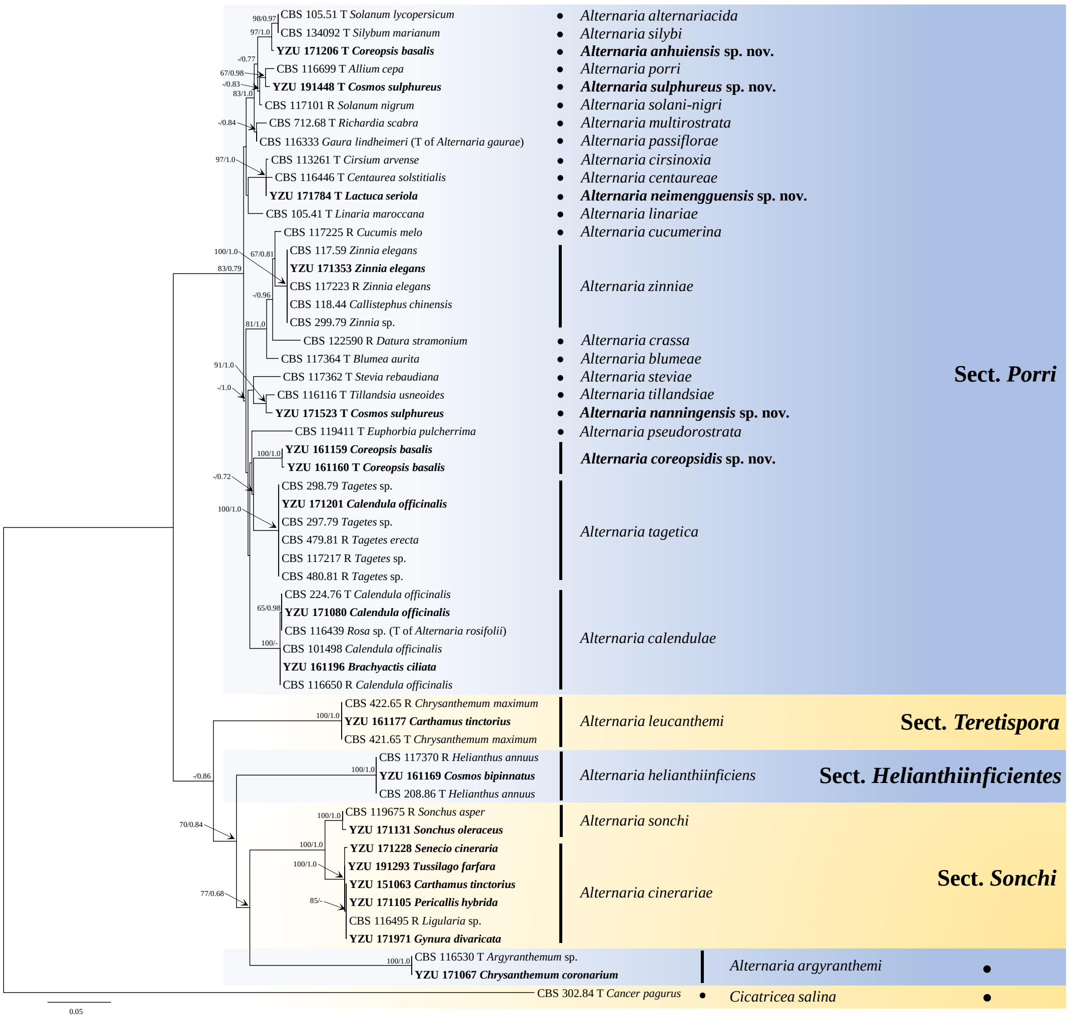

Figure S1. Phylogenetic tree of large-spored *Alternaria* from the Compositae family in China using a maximum likelihood (ML) analysis based on combined GAPDH and RPB2 gene sequences. The RAxML bootstrap support values > 60 % (ML) and Bayesian posterior probabilities > 0.6 (PP) are given at the nodes (ML/PP).
