# Supplementary material for: New Species of Large-Spored Alternaria in Section Porri Associated with Compositae Plants in China
Source: J Fungi (Basel). 2022 Jun 6;8(6):607. doi: 10.3390/jof8060607 (PMC9225545; doi:10.3390/jof8060607)
Supplement: Supplementary file 1 [file jof-08-00607-s001.zip › jof-1752105-Table S1.pdf]

Table S1. The other *Altenraria* species associated with the Compositae plants from China analyzed by phylogeny.

Section/ Monotypic lineage	Species	Strain	Locality	Substrate	GAPDH	RPB2
Porri	*A. calendulae*	**YZU 161196**	**China**	***Brachyactis ciliata*, leaf**	**ON243980**	**ON263326**
		**YZU 171080**	**China**	***Calendula officinalis*, leaf**	**ON243983**	**ON263329**
	A. tagetica	**YZU 171201**	**China**	***C. officinalis*, leaf**	**ON243982**	**ON263328**
	A. zinniae	**YZU 171353**	**China**	***Zinnia elegans*, leaf**	**ON243979**	**ON263325**
Sonchi	*A. cinerariae*	CBS 116495 R	USA	*Ligularia* sp.	KC584109	KC584389
		**YZU 151063**	**China**	***Carthamus tinctorius***	**-**	**MH285937**
		**YZU 171105**	**China**	***Pericallis hybrida***	**-**	**MH285939**
		**YZU 171228**	**China**	***Senecio cineraria***	**MH285947**	**MH285940**
		**YZU 171971**	**China**	***Gynura divaricata***	**MH285948**	**MH285941**
		**YZU 191293**	**China**	***Tussilago farfara***	**-**	**MN584905**
	A. sonchi	CBS 119675 R	Canada	*Sonchus asper*	KC584142	KC584433
		**YZU 171131**	**China**	***Sonchus oleraceus***	**-**	**ON263330**
Teretispora	*A. leucanthemi*	CBS 421.65 T	Netherlands	*Chrysanthemum maximum*	KC584164	KC584472
		CBS 422.65 R	USA	*C. maximum*	KC584165	KC584473
		**YZU 161177**	**China**	***Carthamus tinctorius*, leaf**	**ON243981**	**ON263327**
Helianthiinficientes	*A. helianthiinficiens*	CBS 208.86 T	USA	*Helianthus annuus*	KC584120	KC584403
		CBS 117370 R	UK	*H. annuus*	KC584119	KC584402
		**YZU 161169**	**China**	***Cosmos bipinnatus*, leaf**	**MF414167**	**MH285936**
Monotypic lineage	*A. argyranthemi*	CBS 116530 T	New Zealand	*Argyranthemum* sp.	KC584098	KC584378
		**YZU 171067**	**China**	***Chrysanthemum coronarium***	**MG674139**	**MG647617**
	Cicatricea salina	CBS 302.84 T	North Sea	*Cancer pagurus*	JN383467	KC584450

Note: GenBank accession numbers in bold indicate the newly generated sequences. T: ex-type strain; R: representative strain.
